# Investigating the Role of Gold Nanoparticle Shape and Size in Their Toxicities to Fungi

**DOI:** 10.3390/ijerph15050998

**Published:** 2018-05-16

**Authors:** Kangze Liu, Zhonglei He, Hugh J. Byrne, James F. Curtin, Furong Tian

**Affiliations:** 1 Environmental Sustainability and Health Institute, School of Food Science and Environmental Health, College of Sciences and Health, Dublin Institute of Technology, Cathal Brugha Street, Dublin D08 X622, Ireland; zhonglei.he@mydit.ie (Z.H.); james.curtin@dit.ie (J.F.C.); 2 FOCAS Research Institute, Dublin Institute of Technology, Camden Row, Dublin D08 X622, Ireland; hugh.byrne@dit.ie

**Keywords:** gold nanoparticle, fungi, nanoparticle shape, nanoparticle size, nanotoxicology

## Abstract

Gold nanoparticles (GNPs) are increasingly being used in a wide range of applications, and such they are being released in greater quantities into the environment. Consequently, the environmental effects of GNPs, especially toxicities to living organisms, have drawn great attention. However, their toxicological characteristics still remain unclear. Fungi, as the decomposers of the ecosystem, interact directly with the environment and critically control the overall health of the biosphere. Thus, their sensitivity to GNP toxicity is particularly important. The aim of this study was to evaluate the role of GNP shape and size in their toxicities to fungi, which could help reveal the ecotoxicity of GNPs. *Aspergillus niger*, *Mucor hiemalis*, and *Penicillium chrysogenum* were chosen for toxicity assessment, and spherical and star/flower-shaped GNPs ranging in size from 0.7 nm to large aggregates of 400 nm were synthesised. After exposure to GNPs and their corresponding reaction agents and incubation for 48 h, the survival rates of each kind of fungus were calculated and compared. The results indicated that fungal species was the major determinant of the variation of survival rates, whereby *A. niger* was the most sensitive and *M. himalis* was the least sensitive to GNP exposure. Additionally, larger and non-spherical GNPs had relatively stronger toxicities.

## 1. Introduction

In the growing field of nanotechnology, gold nanoparticles (GNPs) have received a lot of attention, particularly with respect to their potential applications in bio-related areas [1,2,3,4,5]. The unique physical and chemical properties of noble metal nanoparticles such as GNPs, with specific electronic structures different from atoms or bulk states, have long been of interest and have been widely exploited in diversiform areas such as electronics, chemistry, optics, and biomedicine [1,2,3,4,5]. One of the most important characteristics of GNPs is the localized surface plasmon resonance (LSPR). The LSPR phenomena of GNPs are manifest when the dimensions of the GNP are smaller than the extent of the plasmon wavefunction delocalization, resulting in strong resonances of the surface electronic states with radiation in the visible region of the spectrum [6,7]. Although silver nanoparticles show stronger LSPR phenomena, with a stronger absorption, GNPs are more commonly used in biologically-related applications because of their reputed biocompatibility [6,7,8]. Moreover, it is worth mentioning that the potential of GNPs in medical and clinical use has been long investigated [9].

Nevertheless, while silver nanoparticles have been widely used as anti-microbial materials due to their high toxicity [10,11,12], the toxicity of GNPs has not yet been fully understood and has drawn the attention of researchers. Notably, it has been reported that the toxicity of GNPs on microbes depends strongly on the species of microbe and the physicochemical properties of the GNP [13,14,15], and it has also been reported that different shapes of GNPs, such as spheres, rods, triangles, hexagons, prisms, and so on, have different cellular uptake mechanisms and elicit different toxic responses [16,17,18,19]. It has also been reported that the size of GNPs significantly affects the excretion ability of human body, and thus leads to a change in GNP toxicology [9].

With the rapidly increasing applications of GNPs, the possibility of their release into the environment has grown dramatically [20,21,22]. Thus, their effects on the environment, especially their ecotoxicity, have drawn increasing attention [23,24,25]. Therefore, the toxicity of GNPs to organisms that strongly interact with their direct environment, such as fungi and plants, is of critical importance [24,25].

Fungi are found in most terrestrial, marine, and freshwater environments and are the dominant decomposers within the ecosystem. They play a key role in energy cycling and ensuring that nutrients are released from dead and dying plants and animals, thereby ensuring these nutrients remain in circulation within the biosphere [26]. Bioaccumulation of contaminating heavy metals has been observed in terrestrial and water-borne fungi due in part to the large surface area of fungal mycelia, their role as decomposers of dead organic matter, and their ecological niche to extract, concentrate, and recycle nutrients and minerals back into the biosphere [27]. Organisms within an ecosystem that accumulate toxicants are typically more likely to suffer adverse effects at lower environmental concentrations, and thus, the sensitivity of common fungi to GNP exposure is critical, as it may impact negatively on the biosphere’s capacity to recycle organic and inorganic materials. In this study, three kinds of fungi, namely *Aspergillus niger*, *Mucor hiemalis*, and *Penicillium chrysogenum*, are chosen for toxicity assessment, since they are common fungi species, and are widespread in the environment [28,29,30]. Compared to the standard synthesis method of HEPES-reduced GNPs, which only uses HEPES and chloroauric acid [31,32], spherical-shaped GNPs with different sizes are also synthesised by adding different concentrations of monosodium phosphates. Similarly, star/flower-shaped GNPs of various sizes are synthesised by adding different concentrations of disodium phosphates. The toxicities of these GNPs to the three chosen fungi species are examined and compared to determine the role of size and shape in GNP toxicity.

## 2. Materials and Methods

### 2.1. Materials

Hydrogen tetrachloroaurate(III) trihydrate (HAuCl_4_·3H_2_O) was purchased from Fisher Chemical, Ireland. The *N*-2-hydroxyethylpiperazine-*N*-2-ethanesulphonic acid (HEPES) buffer was purchased from Hampton Research (Aliso Viejo, CA, USA). Sodium hydroxide (NaOH), hydrochloride acid (HCl), sodium phosphate monobasic (NaH_2_PO_4_), and disodium hydrogen phosphate (Na_2_HPO_4_) were purchased from Sigma Aldrich (Arklow, Ireland). *Aspergillus niger* ATCC 16404, *Mucor hiemalis* LZB 130, and *Penicillium chrysogenum* LZB 141 strains were purchased from Blades Biological Ltd. (Kent, UK). Potato dextrose agar (PDA) was purchased from Lab M (Lancashire, UK).

Sterilised deionised water, deionised using Elix^®^ Reference Water Purification System from Millipore (Cork, Ireland), and sterilised using Autoclave SX-500E from Mason Technology (Dublin, Ireland), was used for all experiments and solution preparations.

### 2.2. Synthesis of Colloidal GNPs

A 1 mM stock solution of chloroauric acid was made by dissolving hydrogen tetrachloroaurate in water. The 10 mM HEPES buffer stock solution was made by diluting 1 mM HEPES buffer, purchased from Sigma Aldrich (Ireland), with water. Different concentrations of monosodium phosphate and disodium phosphate solutions were prepared by dissolving in water, accordingly.

In the standard synthesis of GNPs, which will be referred to as ‘standard’ in the following sections, 200 µL of 1 mM chloroauric acid were mixed with 200 µL of 10 mM HEPES buffer and 600 µL of water. After 10–15 min, the colour of the solution changed from pale yellow to colourless, and then eventually changed to pink, indicating the formation of GNPs.

In the synthesis of GNPs using phosphates, a similar procedure was followed, with the exception that 200 µL of 1 mM chloroauric acid were mixed with 200 µL of 10 mM HEPES buffer and 600 µL of monosodium phosphate or disodium phosphate solution, of systematically varied concentrations.

For all GNP samples, the mass concentration of gold was 39.394 mg/L.

### 2.3. Characterisation of GNPs

The absorption spectrum of the GNPs in the ultraviolet-visible spectral region was measured using a Perkin Elmer Lambda 900 UV/VIS/NIR Spectrometer. The absorption spectrum was used to monitor the formation of GNPs, and to characterise and discriminate different samples.

A Hitachi SU6600 Field Emission Scanning Electron Microscopy (FESEM) instrument was used to record images of different kinds of GNPs synthesised with or without phosphates. Three representative formulations of GNPs were prepared: (1) standard; (2) with 6 mM of monosodium phosphate; and (3) with 6 mM of disodium phosphate. In the synthesis of GNPs using phosphates, 200 µL of 1 mM chloroauric acid were mixed with 200 µL of 10 mM HEPES buffer and 600 µL of 6 mM of monosodium phosphate or 6 mM of disodium phosphate solution, respectively. After reaction for 1.5 h, the samples were dropped onto silicon wafers and spun for 2.5 min to dry in the air. The samples on silicon substrates were then observed under the scanning electron microscope within 2 days. An accelerating voltage of 20 kV was used for all samples.

The hydrodynamic particle sizes and zeta potentials of different samples were also measured, using a Zetasizer Nano ZS Analyser from Malvern Instruments, Worcestershire, UK.

The pH values of the GNP synthesis systems with different concentrations of monosodium phosphates or disodium phosphates added were measured using Thermo Scientific™ Orion™ 3-Star Benchtop pH Meter (Dublin, Ireland).

### 2.4. Toxicity Test of GNPs on Fungi

The toxicity of the standard GNPs and GNPs synthesised using phosphates was tested. Three species of fungus, *Aspergillus niger*, *Mucor hiemalis*, and *Penicillium chrysogenum* were used in the toxicity test. The fungi samples were prepared by inoculating each in a bottle of sterilised and deionised water and then examined as prepared, and the survival and growth of fungi was measured using the plate count method as described previously [33].

Briefly, for each species of fungus, a control group, a comparison group and a test group were prepared and incubated at the same time. In the control group, each fungus sample was mixed with sterilised deionised water at the ratio of 1:1 and then spread on the potato dextrose agar (PDA) plates. In the comparison group, mixtures of 10 mM HEPES buffer and deionised water or different concentrations of monosodium or disodium phosphates at a ratio of 1:3 were prepared. Then, these mixtures were adjusted using sodium hydroxide and/or hydrochloride acid until the pH values reached 7. Then, the fungus sample was mixed with each mixture at a ratio of 1:1 and each was spread on the PDA plates. In the test group, the GNPs were synthesised using phosphates with the same concentrations as in the comparison group, and the pH values were adjusted to 7 with sodium hydroxide and hydrochloride acid, then centrifuged at 12,000 rpm for 10 min to remove all non-GNP components [34,35]. Liquids were removed after centrifuge, and centrifuged GNP pellets were immediately dispersed with sterilised deionised water to their original volume, then mixed with fungus samples at a ratio of 1:1 and spread on the PDA plates. The GNP exposure dose for all samples was 19.697 mg/L gold. In total, for each kind for fungi, in addition to one control group, eight pairs of comparison groups and test groups were prepared, involving standard GNP, three kinds of GNPs synthesised adding monosodium phosphate, and four kinds of GNPs synthesised adding disodium phosphate.

All chemicals used in the comparison and test groups were sterilised in advance, and triplicates were measured for all test plates mentioned above. All plates were incubated at 28 °C for 48 h, and then the colony-forming units (CFUs) were counted. The CFU results of each comparison and test group were divided by the CFU result of the corresponding control group, indicating the survival rate. During the process of pH adjustment, for all samples, to minimise the change in volume, high concentrations of sodium hydroxide and hydrochloride acid, namely 1 M and 5 M NaOH stock solution and 1 M and 5 M HCl stock solution, were used. For each 3 mL sample solution, less than 20 µL base or acid was added. Samples with base/acid added were shaken for 1 min to mix properly, and then measured with a pH meter. After the pH values were adjusted to 7, samples were sterilised and ready for purification.

In addition, to examine the toxicity of each kind of GNP at different concentrations, further toxicity tests were employed. For each species of fungus, a control group and five test groups were prepared and incubated at the same time.

For control groups, the fungi sample was mixed with sterilised deionised water at a ratio of 1:1, then spread on PDA plates. In five test groups, the fungi sample was mixed with five different concentrations of each kind of GNP at ratio of 1:1, and then spread on PDA plates for incubation.

Apart from the original concentration of each GNP (19.697 mg/L gold), all eight kinds of GNPs were concentrated and diluted 20 and 5 times after pH adjustment to 7, creating four more concentrations: 393.940 mg/L, 98.485 mg/L, 3.939 mg/L, and 0.985 mg/L gold. The colloidal GNPs at original concentrations were centrifuged at 12,000 rpm for 10 min to concentrate, and diluted using sterilised deionised water accordingly.

All these tests were repeated four times, and the samples were incubated at 28 °C for 48 h. CFU results of the test groups were divided by CFU results of the control groups accordingly, indicating survival rates.

No colour change of the GNP dispersions was observed after the adjustment of pH and purification, nor after plating on the culture media. It was therefore assumed that the physico-chemical characteristics of the NP dispersions were not changed significantly for the toxicity testing.

### 2.5. Statistical Analysis

A paired *t*-test with two-tailed *p* value and 95% confidence interval was employed to evaluate the difference between the survival rates of the comparison group and the experimental group for the evaluation of toxicity. Two-way ANOVA using the Tukey test and 95% confidence interval was employed to evaluate the role of GNP size on GNP toxicities. These two statistical analyses and all curve fittings were carried out using the software GraphPad Prism 7 (La Jolla, CA, USA).

Three-way ANOVA was employed to evaluate the role of fungi species and GNP shape on GNP toxicities. This analysis and the plotting of survival rates was carried out using the software JMP.

Detailed data from Figures 2–5, including all values of mean, Standard error of the mean (SEM) and numbers of replicates, are presented in Supplementary Material Tables S1–S4 accordingly.

## 3. Results

### 3.1. Characterisation of GNPs

The measured physicochemical characteristics of each kind of GNP are shown in Table 1.

For GNPs synthesised by adding monosodium phosphates, as the concentration of monosodium phosphate increases, the absolute value of zeta potential increases. In contrast, for GNPs synthesised by adding disodium phosphates, the absolute value of zeta potential decreases as the concentration of disodium phosphate increases.

The average hydrodynamic diameter of standard GNP was found to be ~62 nm, using dynamic light scattering (DLS). GNPs synthesised by adding monosodium phosphate reach the smallest size of ~4.6 nm in diameter when 6 mM monosodium phosphate are added, and higher or lower concentrations of phosphate lead to larger particles. The diameters of GNPs synthesised by adding disodium phosphates remain smaller than 53 nm when the concentration of phosphate is lower than 100 mM, and particle sizes grow larger with the increase of disodium phosphate concentration after that.

**Figure 1:**
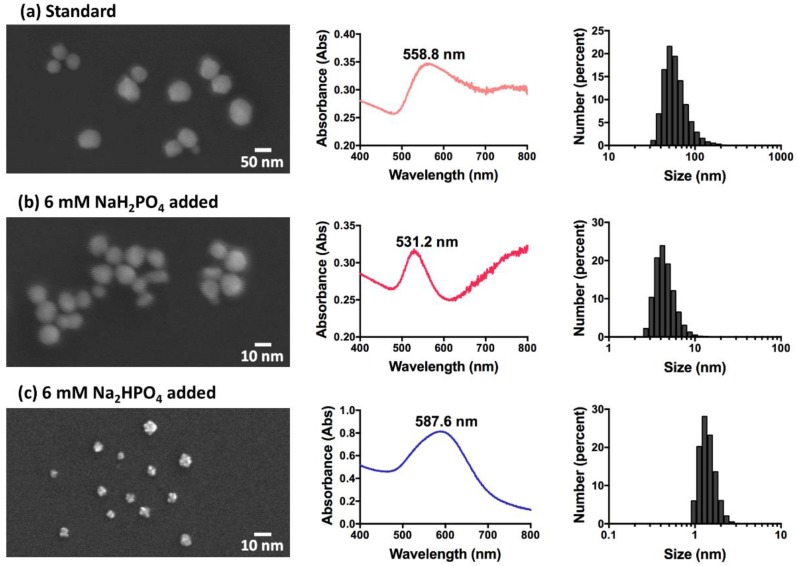
Field Emission Scanning Electron Microscopy (FESEM) images of GNPs with different sizes and shapes, and the corresponding UV-VIS spectra and size number distribution histogram (from left to right). (**a**) Standard GNPs synthesised with only chloroauric acid and HEPES buffer; (**b**) GNPs synthesised with chloroauric acid, HEPES buffer, and 6 mM monosodium phosphate; (**c**) GNPs synthesised with chloroauric acid, HEPES buffer, and 6 mM disodium phosphate. HEPES: *N*-2-hydroxyethylpiperazine-*N*-2-ethanesulphonic acid.

The shape and size differences of the GNPs were analysed under FESEM. The standard and those synthesised with monosodium phosphate are all spheroidally-shaped, as shown in Figure 1a,b. However, even though they are both gold nanospheres, the diameter of the standard GNPs is about 30–100 nm, while the diameter of those synthesised with 6 mM monosodium phosphate is much smaller, ~2–10 nm. The GNPs synthesised by adding higher concentrations of monosodium phosphate tend to aggregate into large clusters, which results in a UV-VIS peak at 800 nm, and a large scattering background to the UV-VIS spectra.

In Figure 1c, it can be inferred that the GNPs synthesised with 6 mM disodium phosphate are star-shaped or flower-shaped, which explains the phenomenon that, although most GNPs with an LSPR peak of more than 580 nm and a blue colour are aggregated or large-sized, these appear to be rather smaller, with diameters of only 1–3 nm.

### 3.2. Toxicity Test of GNPs with Different Sizes and Shapes at Concentration of 19.697 mg/L Gold

**Figure 2:**
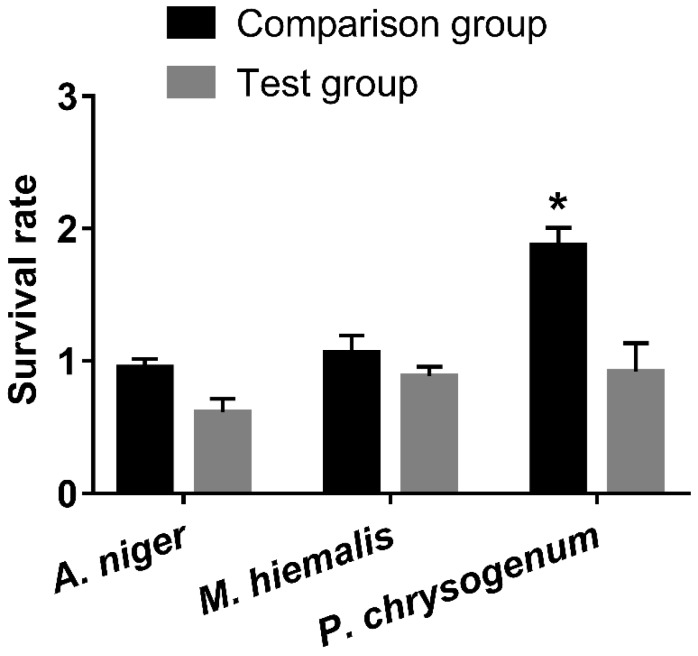
Survival rates of *Aspergillus niger*, *Mucor hiemalis*, and *Penicillium chrysogenum* in the presence of standard GNP and the corresponding comparison group. All comparison group experiments were replicated three times, and test group experiments were replicated seven times. All data points presented are shown as mean ± SEM. The statistical significance was assessed by paired *t*-test with two-tailed *p*-value and a 95% confidence interval (* *p* < 0.05). SEM: Standard error of the mean.

After mixing pH-adjusted HEPES and standard GNP with *A. niger*, *M. hiemalis*, and *P. chrysogenum*, and incubating for 48 h, the fungal growth on each plate was compared by calculating relative survival rates (Figure 2). It can be seen that the survival rates of comparison groups were all around or above 1 for all three species of fungus, which indicated that HEPES was not inhibiting the growth. On the other hand, the survival rates of fungi in the presence of standard GNP were decreased, which indicated that standard GNP (19.697 mg/L gold) inhibited the growth of the fungi, among which *P. chrysogenum* was the least affected with the highest survival rate, followed by *M. hiemalis*, and *A. niger* was inhibited the most.

**Figure 3:**
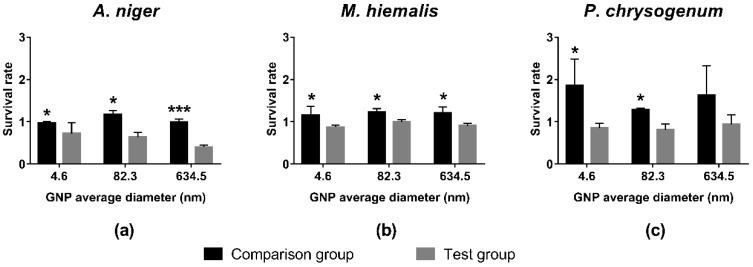
Survival rates of (**a**) *A. niger*; (**b**) *M. hiemalis*; and (**c**) *P. chrysogenum* in the presence of spherical GNPs synthesised with monosodium phosphates, and the corresponding comparison groups. All comparison group experiments were replicated three times, and test group experiments were replicated seven times. All data points presented are shown as mean ± SEM. The statistical significance was assessed by paired *t*-test with two-tailed *p* value and 95% confidence interval (* *p* < 0.05, *** *p* < 0.001).

In Figure 3, it can be seen that survival rates of comparison groups were greater than 1, which indicates that the mixture of HEPES and monosodium phosphates promotes the growth of fungi, albeit to differing extents. Similar to the effect caused by HEPES only, the growth of *P. chrysogenum* was increased the most, followed by that of *M. hiemalis*, and finally, *A. niger*.

In contrast, the survival rates of test groups were all decreased to below 1, which indicated that GNPs synthesised using monosodium phosphates with a concentration of 19.697 mg/L GNP inhibited the fungi growth. Among the three fungi species, the growth of *A. niger* was inhibited most significantly with lowest survival rates, and a trend is shown of larger GNPs having stronger inhibition effects (Figure 3a). In comparison, *M. hiemalis* and *P. chrysogenum* showed less sensitivity to GNPs, and no statistically significant size trend was observed. However, the average survival rates of the test groups of these two fungi were still lower than those of the corresponding comparison groups.

**Figure 4:**
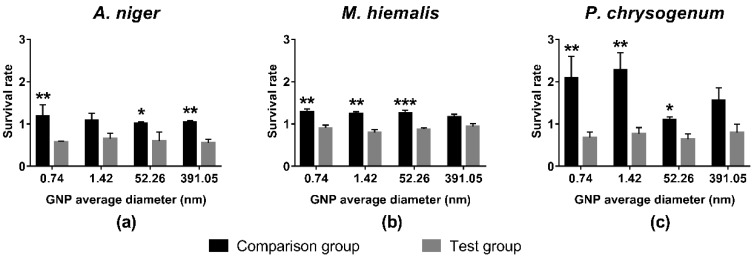
Survival rates of (**a**) *A. niger*; (**b**) *M. hiemalis*; and (**c**) *P. chrysogenum* in the presence of star/flower-shaped GNPs synthesized with disodium phosphates, and the corresponding comparison groups. All comparison group experiments were replicated three times, and test group experiments were replicated seven times. All data points presented are shown as mean ± SEM. The statistical significance was assessed by paired *t*-test with two-tailed *p* value and 95% confidence interval (* *p* < 0.05, ** *p* < 0.01, *** *p* < 0.001).

Similar to HEPES and mixtures of HEPES and monosodium phosphates, the mixtures of HEPES and disodium phosphates promoted the growth of all three kinds of fungus, as survival rates of all comparison groups were above 1 (Figure 4). Among the three fungi species, the growth of *P. chrysogenum* was promoted the most, while *A. niger* and *M. hiemalis* reacted less sensitively.

On the other hand, the survival rates of all test groups were below 1, indicating that these GNPs synthesised by adding disodium phosphates inhibited the growth of all three kinds of fungi when the concentration was 19.697 mg/L GNP. *M. hiemalis* exhibited the least sensitivity to GNPs, having survival rates only slightly lower than 1 (Figure 4b), followed by *P. chrysogenum* (Figure 4c), and the growth of *A. niger* was inhibited most, resulting in the lowest survival rates (Figure 4a). No significant size trend was observed in these groups.

Results of *t*-tests between survival rates of the comparison group and test groups are indicated in Figure 2, Figure 3 and Figure 4. Most pairs of comparison and test groups showed significant differences. Even for those pairs that did not show significant differences, the survival rates of the test groups were still lower than for the corresponding comparison groups.

In general, HEPES, monosodium phosphate, and disodium phosphate caused large increases in growth for *P. chrysogenum*, small increases for *M. hiemalis*, and had only a small influence on *A. niger*. In contrast, GNPs at concentration of 19.697 mg/L gold inhibited the growth of fungi to different extents. *M. hiemalis* reacted least sensitively to the inhibition of GNPs, followed by *P. chrysogenum*, and *A. niger* was inhibited the most. Different sizes and shapes of GNPs caused different inhibition results, whereby the overall inhibition caused by star/flower-shaped GNPs was slightly stronger than spherical GNPs, and larger spherical GNPs elicited stronger inhibition of *A. niger*.

### 3.3. Dose–Response Curves

**Figure 5:**
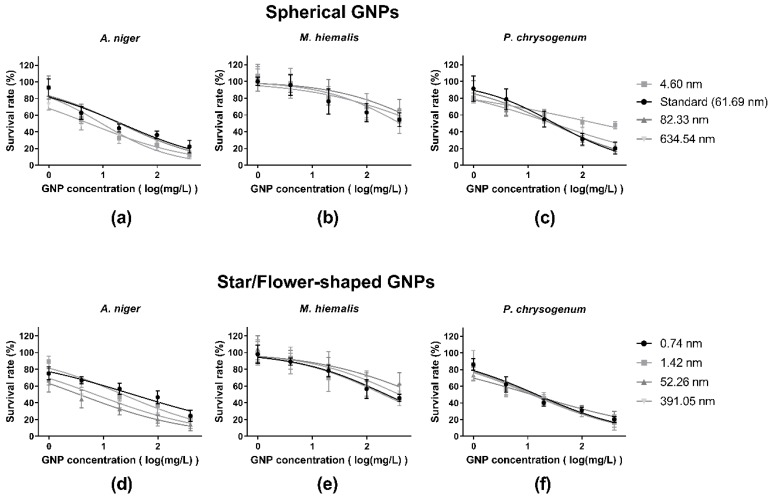
Dose–response curves of *A. niger*, *M. hiemalis*, and *P. chrysogenum* to different sizes of spherical GNPs (**a**–**c**, respectively) and star/flower-shaped GNPs (**d**–**f**, respectively). All experiments were performed in four replicates. All data points presented are shown as mean ± SEM.

**Figure 6:**
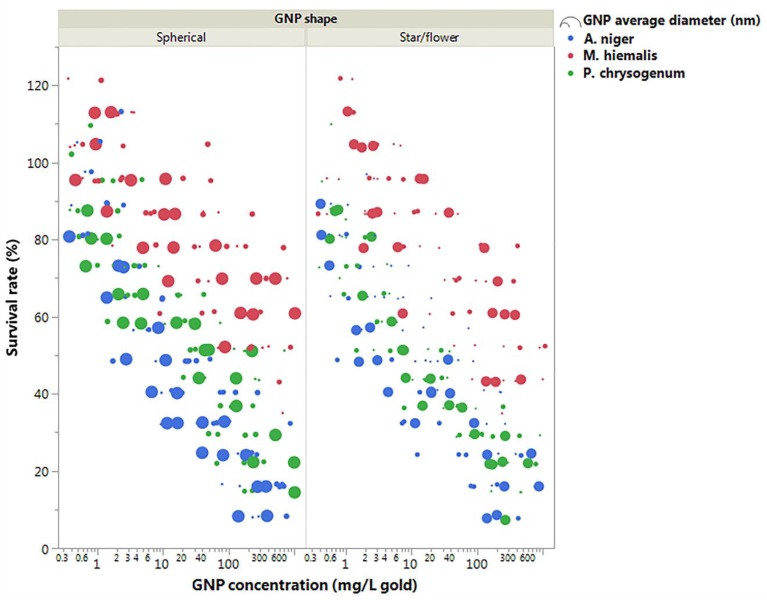
Plot figure of survival rates of fungi in the presence of different GNPs. As indicated in the legend, the size of the markers represents the size of GNPs, where larger markers represent larger GNPs, and the colour of the markers indicates fungi species.

The response of each kind of fungus to the presence of five concentrations of each kind of GNP was tested. (Figure 5 and Figure 6).

Although the survival rates of *M. hiemalis* decreased in the presence of higher concentrations of standard GNPs, the response did not fit well to a standard dose–response curve. With this exception, all groups are well fitted by standard dose–response curves.

By comparing Figure 5a,d, b,e, c,f, it can be seen that the two shapes of GNPs did not elicit markedly different toxic responses from each kind for fungus. The dose–response curves indicate that different sizes of GNP caused differences in GNP toxicities, and the grades of differences varied according to GNP shape and fungi species, among which *A. niger* responded most sensitively to the size change of star/flower-shaped GNPs (Figure 5d).

From Figure 6 it can be seen that the major contribution to the differences of GNP toxicity was caused by fungi species. *M. hiemalis* responded least sensitively to the presence of all kinds of GNPs, *A. niger* had slightly higher sensitivity than *P. chrysogenum* to spherical GNPs exposure (Figure 6 left panel) and large star/flower-shaped GNPs (Figure 6 right panel), and *P. chrysogenum* was more sensitive to small star/flower-shaped GNPs (Figure 6 right panel). It was also shown in Figure 6 that, for *A. niger* and *P. chrusogenum*, larger GNPs decreased the survival rates more significantly compared to smaller GNPs.

The IC_50_ values and 95% confidence intervals of each dose–response curve are shown in Table 2. The overall IC_50_ values of all eight kinds of GNPs on *M. hiemalis* were much higher than those of the other two fungi, indicating again that *M. hiemalis* responded the least to the toxicities of GNPs, followed by *P. chrysogenum*, and *A. niger* responded most sensitively to GNPs, yielding the smallest IC_50_ values.

For *P. chrysogenum*, it can be seen that all IC_50_ values for star/flower-shaped GNPs were lower than those for spherical GNPs, indicating that star/flower-shaped GNPs elicited stronger toxic responses in *P. chrysogenum* than spherical GNPs. Comparing IC_50_ values of same shaped GNPs with different sizes, it can be seen that smaller GNPs elicited weaker toxic responses in *P. chrysogenum*.

In contrast, trends are different for *A. niger*. While there were no significant differences between the toxicities of spherical and star/flower-shaped GNPs, the trend of size varied as well. Although star/flower-shaped GNPs held same trend that larger GNPs had stronger toxicities on *A. niger*, it can be seen that for spherical GNPs, both largest and smallest GNPs held stronger toxicities than medium-sized GNPs.

### 3.4. Statistical Analysis Results

To examine the role of GNP size on GNP toxicity, two-way ANOVA was employed using the dose–response data points of two shapes of GNPs on each fungi species. GNP size and GNP concentration has been used as factors influencing fungal survival rates. The results are listed in Table 3.

Apart from *M. hiemalis*, which reacted the least sensitively to GNPs, the results showed that the size of GNP influenced fungi survival rates significantly for both GNP shapes in the case of *A. niger*, and for spherical-shaped GNP in the case of *P. chrysogenum*. Thus, it was confirmed that the size of GNPs was a factor in determining GNP toxicities, although it still depended on GNP shape and fungi species. For most groups, no significant two-way correlation was observed between observed toxicity and GNP size and/or GNP concentration, except for *A. niger* with star/flower-shaped GNPs and *P. chrysogenum* with spherical GNPs.

Three-way ANOVA was also employed, and run on all 480 data points of fungi survival rate to examine the effect of fungi species, GNP shape and GNP concentration on GNP toxicities (Table 4). The results showed that all three factors had significant influence on the variation of fungi survival rates. The *p* value of GNP concentration is less than 0.0001, which is expected, since most data points fit well to the dose–response curves. The *p* values of GNP shape and fungi species proved that these two were both factors that caused significant variation of GNP toxicities. The results also showed that there were no significant two-way or three-way correlations between any of these three factors.

## 4. Discussion

### 4.1. Ensuring Reproducibility of GNP Mass Concentrations

Various methods for the synthesis of GNP have been developed with limited knowledge of potential environmental toxicity. In our study, we assessed toxicity in fungi using GNPs synthesised by three different methods: standard GNP, GNPs synthesised adding monosodium phosphates, and GNPs synthesised using disodium phosphates. In order to accurately compare toxicities of GNP produced using the three methods, we needed to ensure the reproducibility of mass concentration of GNP produced. All samples, whether with phosphates added or not, were synthesised by mixing 200 µL of 1 mM chloroauric acid and 200 µL of 10 mM HEPES buffer for every 1 mL sample.

The HEPES-reduced GNP synthesis method has been employed and studied for a decade, and it has been reported that the difference of the molar ratio of chloroauric acid and HEPES strongly affects the characteristics of the GNP synthesised [31]. In this study, a molar ratio of 1:10 between chloroauric acid and HEPES was used in all syntheses, which ensured the complete reduction of chloroauric acid into atomic gold. Thus, the mass concentration of gold for all GNP samples is the same, which allows their toxicities to be compared since the volumes of GNPs mixed with all fungi samples are the same, leading to same exposure dose as 19.697 mg/L for all samples.

The LSPR peaks of the GNPs synthesised using phosphates are markedly different to those of the standard GNP (Table 1). As reported by Haiss et al., the size and concentration of GNPs within the size range of 5–100 nm can be directly determined from UV-VIS spectra according to corrected Mie theory [36]. While GNPs with average hydrodynamic diameter ~62 nm and LSPR peak of 558.8 nm can be synthesised using a typical standard protocol, GNPs sized ~4–85 nm can be synthesised when adding 2–6 mM monosodium phosphate, and GNPs sized 0.7–55 nm can be synthesised when 1–50 mM disodium phosphate is added (Table 1). For these GNP samples, the smaller the diameters, the lower the LSPR peak wavelengths according to corrected Mie theory [36].

Different shapes of GNPs were formed with or without phosphate added (Figure 1). The difference in morphology was caused by different ion levels in reaction systems. HEPES, as the reducer of Au (III), was also the directing agent of GNP shape and size [37,38,39]. Since pH plays an important role in HEPES-reduced GNP synthesis [31], the addition of different concentrations of phosphates changed the reaction pH, thus causing synthesis of GNPs with different sizes and shapes. HEPES free radicals were created while Au (III) was reduced to Au (0), and assembled on Au (0) atoms during the formation of GNPs [32,40]. The HEPES free radicals assembled on GNPs varied in quantity, and may have also had phosphate ions attached with the addition of different phosphates, which caused the morphology change of GNPs synthesised.

### 4.2. Confirming Toxicity of GNPs

As shown in Figure 2, Figure 3 and Figure 4, exposure of the fungi to HEPES, monosodium phosphate or disodium phosphate did not cause a decrease in fungi growth. In contrast, they promoted the growth of fungi to different extents, whereby *P. chrysogenum* reacted the most sensitively.

HEPES has long been used as a buffer in biology and biochemistry, especially in culturing, since it has high biocompatibility [41]. It has been reported that the phosphate is essential for the growth of mould fungi, including *Aspergillus*, *Penicillium*, and *Rhizopus* [42], and increases the cellular activities of *M. hiemalis* [43]. Thus, increased growth of fungi in the presence of HEPES and phosphates is to be expected. In contrast, all survival rates of test groups decreased in the presence of GNPs. Since the GNPs were purified before mixing with fungi samples, the only source that caused the decrease was GNPs and the compounds assembled on them.

### 4.3. Comparing the Role of Fungi Species and GNP Size and Shape on GNP Toxicity

Survival rates of fungi exposed different concentrations of each GNP were examined. As shown in Table 4, the change of GNP concentration caused significant variation to the survival rates. Thus, dose–response curve fittings were employed to all data sets (Figure 5), and IC_50_ values were calculated (Table 2).

#### 4.3.1. Fungi Species

Fungi species was found to be the main factor governing the toxic response, with the most significant *p* value result (Table 4). In Figure 5 and Figure 6, it can be seen that *M. hiemalis* reacted least sensitively to GNP exposure, followed by *P. chrysogenum*, which in turn is more sensitive than *A. niger*.

The difference in sensitivity to GNP exposure may be caused by the different accumulation abilities of these three kinds of fungi. The capacity of fungi for heavy metal removal has long been recognised and utilised [44,45]. It has also been reported that the metal accumulation ability varies significantly between different species [46,47,48]. *Aspergillus* has been reported to have higher accumulation capacity than *Penicillium* for many metals such as cobalt, chromium, copper, cadmium, and nickel [49], and *A. niger* has been reported to have especially high accumulation capacity [50]. Meanwhile, *Mucor* was reported to lower the capacity for accumulation of some metals like copper [51]. The difference in GNP toxicity sensitivities may be caused by higher accumulation of GNPs by *A. niger*, while *P. chrysogenum* and *M. hiemalis* accumulated fewer. On the other hand, the tolerance to metal toxicity varies significantly among fungi species, and also depends on other factors like pH and cationic activation [52,53]. The low sensitivity of *M. hiemalis* and high sensitivity of *A. niger* to GNPs may also be caused by different intrinsic tolerances.

#### 4.3.2. GNP Size

The size of GNPs was a significant factor for the responses of *A. niger* and *P. chrysogenum* (Table 3). Since *M. hiemalis* had little response to the presence of GNPs, the GNP size effects are only discussed in the context of *A. niger* and *P. chrysogenum*. With the exception of the low IC_50_ value of *A. niger* exposed to 4.60 nm spherical GNP, which could be caused by experimental error, it appeared that smaller GNPs elicited stronger toxicity in both *A. niger* and *P. chrysogenum* (Figure 5, Figure 6 and Table 2).

In general, after being absorbed and accumulated by fungi, larger GNPs had stronger toxicities than smaller GNPs. This may be because larger GNP have more HEPES free radicals assembled on their surfaces [32,40], which causes damage to the fungi and thus reduces the survival rates. It has been reported that, while HEPES in itself is non-mutagenic, the HEPES free radicals created in the presence of Au(III) cause severe DNA damage and subsequent mutations [54]. During the synthesis process, HEPES free radicals were created and assembled on GNPs, which are not removed during purification. After mixing with fungi samples, these free radicals can be absorbed by fungi along with GNPs, and cause damage to fungal DNA, and subsequent death. Larger GNPs, with larger surface area, have more HEPES free radicals assembled on their surface, leading to more DNA damage and higher toxicities.

#### 4.3.3. GNP Shape

The three-way ANOVA results showed that GNP shape was also a significant factor in determining fungi survival rates (Table 4). In comparison, the overall survival rates of fungi in the presence of star/flower-shaped GNPs were lower than spherical GNPs, indicating the slightly higher toxicities (Figure 5, Figure 6 and Table 2). A similar phenomenon has been reported, whereby non-spherical GNPs have stronger cytotoxic effects on Calu-3 epithelial cells than spherical GNPs [16]. This may be because relatively more HEPES free radicals were carried by star/flower-shaped GNPs than spherical GNPs, since they have larger specific surface area. Alternatively, the shape may influence the internalisation and therefore accumulation rates.

However, compared to the other two factors, GNP shape was only a minor factor in GNP toxicities with larger *p* value, and examinations on toxicities of same sized GNPs in different shapes are needed.

## 5. Conclusions

The role of shape and size of GNPs on their toxicities on three kinds of fungi, *A. niger*, *M. hiemalis*, and *P. chrysogenum*, were investigated. Two kinds of GNP shape (spherical and star/flower-like shaped) with different sizes were examined. The spherical-shaped GNPs were synthesised using two methods: the standard synthesis method, which creates standard GNPs with an average hydrodynamic diameter of 62 nm; and the synthesis method adding different concentrations of monosodium phosphates, which creates GNPs sized between 4.6 nm and 85 nm and large aggregates with a size of 635 nm. The star/flower-shaped GNPs were synthesised by adding different concentrations of disodium phosphates and were sized between 0.7 nm and 400 nm.

It has been found that while HEPES and phosphates promote the growth of fungi to different extents, GNPs decrease the survival rates.

Fungi species is the major influencing factor in the variation of GNP toxicities, due to the differences in accumulation capacity. *A. niger* was the most sensitive, followed by *P. chrysogenum*, while *M. hiemalis* was only slightly affected.

Larger GNPs and non-spherical GNPs appeared more toxic to fungi because they were able to carry more HEPES free radicals into fungi cells, causing DNA damage and mutations.

A more complete understanding of the detailed mechanism of toxicity could be facilitated by visualisation of the uptake and trafficking of the GNPs by fungi. It is anticipated that this will be investigated in a follow-on study.

## Figures and Tables

**Table 1:** Physicochemical characteristics of the gold nanoparticles (GNPs) synthesised using different phosphates. LSPR: localized surface plasmon resonance.

Sample ^1^	Shape	Concentration of Phosphate Added (mM)	LSPR Peak (nm)	Diameter (nm ± SD)	Zeta Potential (mV ± SD)	Average Equivalent Spherical Total Surface Area ^2^ Per 1 mL (cm^2^)
Standard	Spherical	0	558.8	61.69 ± 22.35	−23.50 ± 0.21	1.98
NaH_2_PO_4_ added	Spherical	2	565.0	82.33 ± 35.86	−26.43 ± 0.13	1.49
		6	531.2	4.60 ± 1.32	−29.67 ± 0.13	26.60
		40	532.2	634.54 ± 224.30	−36.87 ± 0.45	0.19
Na_2_HPO_4_ added	Star-shaped/Flower-shaped	1	625.8	52.26 ± 14.94	−40.80 ± 0.24	2.34
		6	587.6	1.42 ± 0.32	−34.97 ± 0.35	86.09
		50	543.4	0.74 ± 0.25	−23.73 ± 0.60	165.29
		240	664.0	391.05 ± 153.47	−22.13 ± 0.27	0.31

^1^ Sample refers to the GNP synthesis methods. “Standard” refers to the GNPs synthesised using the standard synthesis protocol. “NaH_2_PO_4_ added” and “Na_2_HPO_4_ added” refers to the GNPs synthesised adding different concentrations of monosodium and disodium phosphates accordingly; ^2^ Average total surface areas per 1 mL GNP presented were calculated assuming spherical GNPs. The effective surface area of the star/flower-shaped GNPs may be significantly higher than listed, depending on the relative size of any molecular species with which they interact.

**Table 2:** IC_50_^1^ values and 95% confidence intervals of dose–response curves.

GNP Shape	Diameter Mean	*A. niger*		*M. hiemalis*		*P. chrysogenum*	
		IC_50_	95% Confidence Interval	IC_50_	95% Confidence Interval	IC_50_	95% Confidence Interval
Spherical	61.69 (Standard)	21.55	13.34–34.80	N/A ^2^	N/A	30.39	20.04–46.09
	4.60	9.46	6.242–14.34	408.61	168.0–994.1	177.25	102.9–305.3
	82.33	20.40	12.62–32.97	1077.59	220.5–5267	26.51	18.15–38.71
	634.54	5.54	3.928–7.814	950.40	269.3–3354	27.82	18.92–40.91
Star-shaped/Flower-shaped	0.74	34.42	21.69–54.62	224.66	140.2–360.0	14.58	10.15–20.94
	1.42	22.42	15.10–33.30	196.65	94.35–409.9	13.16	7.729–22.40
	52.26	3.56	2.078–6.114	904.56	214.7–3810	11.92	8.335–17.05
	391.05	7.01	3.947–12.44	391.57	155.1–988.4	11.65	8.073–16.80

^1^ IC_50_: Half maximal inhibitory concentration. ^2^ N/A: Not applicable.

**Table 3:** Two-way ANOVA analysis results on fungi survival rates using GNP size and GNP concentration as factors.

Source of Variation	Analysis Subject	*A. niger*		*M. hiemalis*		*P. chrysogenum*	
		Spherical GNP	Star/Flower-Shaped GNP	Spherical GNP	Star/Flower-Shaped GNP	Spherical GNP	Star/Flower-Shaped GNP
GNP size	*F* value	14.82	29.85	2.099	2.183	9.069	0.2945
	*p* value	<0.0001	<0.0001	0.1137	0.1031	<0.0001	0.8292
	*p* value summary ^2^	****	****	ns	ns	****	ns
GNP size × GNP concentration ^1^	*F* value	1.397	1.99	0.7349	0.5058	6.309	1.131
	*p* value	0.2029	0.0481	0.7103	0.8999	<0.0001	0.3606
	*p* value summary ^2^	ns	*	ns	ns	****	ns

^1^ GNP size × GNP concentration: the interaction between GNP size and GNP concentration. ^2^ ns: *p* ≥ 0.05, * *p* < 0.05, **** *p* < 0.0001.

**Table 4:** Three-way ANOVA analysis results on fungi survival rates using GNP shape, concentration, and fungi species as factors.

Source of Variation	Degrees of Freedom	*F* Value	*p* Value	*p* Value Summary ^2^
GNP shape	1	11.1958	0.0009	***
Fungi species	2	184.7116	<0.0001	****
GNP concentration	1	447.8957	<0.0001	****
GNP shape × Fungi species ^1^	2	2.7142	0.0673	ns
GNP shape × GNP concentration	1	0.1539	0.6950	ns
Fungi species × GNP concentration	2	1.1037	0.3325	ns
GNP shape × Fungi species × GNP concentration	2	0.6936	0.5003	ns

^1^ × represents the interaction between factors. ^2^ ns: *p* ≥ 0.05, *** *p* < 0.001, **** *p* < 0.0001.

## Supplementary Materials

The following are available online at http://www.mdpi.com/1660-4601/15/5/998/s1, Table S1: Mean, SEM and number of replicates for data points in Figure 2, Table S2: Mean, SEM and number of replicates for data points in Figure 3, Table S3: Mean, SEM and number of replicates for data points in Figure 4, Table S4: Mean, SEM and number of replicates for data points in Figure 5.
