# Supplementary material for: Investigating the Role of Gold Nanoparticle Shape and Size in Their Toxicities to Fungi
Source: Int J Environ Res Public Health. 2018 May 16;15(5):998. doi: 10.3390/ijerph15050998 (PMC5982037; doi:10.3390/ijerph15050998)
Supplement: Supplementary file 1 [file ijerph-15-00998-s001.pdf]

Supplementary Material

Table S1. Mean, SEM and number of replicates for data points in Figure 2.

Fungi species	Group	Mean	SEM	Number of replicates
A. niger	Comparison group	0.95	0.06	3
	Test group	0.61	0.10	7
M. hiemalis	Comparison group	1.06	0.13	3
	Test group	0.88	0.07	7
P. chrysogenum	Comparison group	1.87	0.13	3
	Test group	0.92	0.21	7

Table S2. Mean, SEM and number of replicates for data points in Figure 3.

Fungi species	GNP average diameter (nm)	Group	Mean	SEM	Number of replicates
A. niger	4.6	Comparison group	0.97	0.03	3
		Test group	0.72	0.26	7
	82.3	Comparison group	1.18	0.09	3
		Test group	0.64	0.11	7
	634.5	Comparison group	0.99	0.07	3
		Test group	0.40	0.05	7
M. hiemalis	4.6	Comparison group	1.16	0.20	3
		Test group	0.87	0.04	7
	82.3	Comparison group	1.23	0.08	3
		Test group	1.00	0.05	7
	634.5	Comparison group	1.21	0.14	3
		Test group	0.91	0.06	7
P. chrysogenum	4.6	Comparison group	1.86	0.62	3
		Test group	0.85	0.12	7
	82.3	Comparison group	1.29	0.03	3
		Test group	0.81	0.13	7
	634.5	Comparison group	1.62	0.70	3
		Test group	0.94	0.22	7

Table S3. Mean, SEM and number of replicates for data points in Figure 4.

Fungi species	GNP average diameter (nm)	Group	Mean	SEM	Number of replicates
A. niger	0.74	Comparison group	1.18	0.28	3
		Test group	0.58	0.02	7
	1.42	Comparison group	1.09	0.16	3
		Test group	0.65	0.13	7
	52.26	Comparison group	1.02	0.03	3
		Test group	0.59	0.22	7
	391.05	Comparison group	1.04	0.03	3
		Test group	0.56	0.08	7
M. hiemalis	0.74	Comparison group	1.29	0.07	3
		Test group	0.91	0.07	7
	1.42	Comparison group	1.24	0.05	3
		Test group	0.80	0.07	7
	52.26	Comparison group	1.26	0.07	3
		Test group	0.88	0.04	7
	391.05	Comparison group	1.17	0.06	3
		Test group	0.94	0.06	7

P. chrysogenum	0.74	Comparison group	2.09	0.50	3
		Test group	0.68	0.13	7
	1.42	Comparison group	2.28	0.40	3
		Test group	0.77	0.15	7
	52.26	Comparison group	1.10	0.07	3
		Test group	0.64	0.12	7
	391.05	Comparison group	1.56	0.30	3
		Test group	0.80	0.20	7

Table S4. Mean, SEM and number of replicates for data points in Figure 5.

GNP shape	Fungi species	GNP concentration (mg/L)	GNP average diameter (nm)											
			61.69 nm (Standard)			4.60 nm			82.33 nm			634.54 nm		
			Mean	SEM	n	Mean	SEM	n	Mean	SEM	n	Mean	SEM	n
Spherical	*A. niger*	393.94	22.30	3.88	4	12.16	2.34	4	16.22	3.31	4	12.16	2.34	4
		98.49	36.49	2.34	4	24.32	3.31	4	36.49	2.34	4	26.35	2.03	4
		19.70	44.59	2.34	4	32.43	3.31	4	46.62	2.03	4	34.46	2.03	4
		3.94	62.84	3.88	4	56.76	3.31	4	62.84	5.10	4	48.65	3.31	4
		0.98	93.24	5.23	4	93.24	5.23	4	93.24	7.02	4	72.97	3.31	4
	M. hiemalis	393.94	54.35	4.16	4	52.17	7.10	4	67.39	5.47	4	63.04	2.17	4
		98.49	63.04	5.47	4	69.57	7.94	4	69.57	7.94	4	67.39	5.47	4
		19.70	76.09	7.43	4	80.43	4.16	4	91.30	5.61	4	82.61	5.61	4
		3.94	95.65	6.15	4	97.83	8.96	4	97.83	5.47	4	86.96	3.55	4
		0.98	100.00	2.51	4	106.52	5.47	4	104.35	7.94	4	106.52	4.16	4
	P. chrysogenum	393.94	20.12	3.50	4	47.56	2.11	4	21.95	2.99	4	21.95	2.99	4
		98.49	31.10	3.50	4	51.22	2.99	4	29.27	2.99	4	43.90	2.99	4
		19.70	54.88	4.72	4	62.20	2.11	4	58.54	2.99	4	54.88	2.11	4
		3.94	78.66	6.25	4	71.34	1.83	4	65.85	2.99	4	62.20	2.11	4
		0.98	91.46	7.62	4	80.49	2.99	4	91.46	4.72	4	80.49	2.99	4

GNP shape	Fungi species	GNP concentration (mg/L)	GNP average diameter (nm)											
			0.74 nm			1.42 nm			52.26 nm			391.05 nm		
			Mean	SEM	n	Mean	SEM	n	Mean	SEM	n	Mean	SEM	n
Star/Flower-shaped	*A. niger*	393.94	24.32	3.31	4	22.30	2.03	4	14.19	3.88	4	16.22	3.31	4
		98.49	46.62	3.88	4	36.49	5.23	4	20.27	2.34	4	26.35	6.92	4
		19.70	56.76	3.31	4	46.62	2.03	4	32.43	3.31	4	40.54	3.31	4
		3.94	66.89	2.03	4	64.86	3.31	4	44.59	5.23	4	48.65	3.31	4
		0.98	75.00	3.88	4	89.19	3.31	4	66.89	6.92	4	75.00	6.92	4
	M. hiemalis	393.94	45.65	2.17	4	43.48	3.55	4	63.04	6.52	4	52.17	5.02	4
		98.49	56.52	5.61	4	58.70	6.52	4	67.39	4.16	4	63.04	7.43	4
		19.70	78.26	3.55	4	69.57	7.94	4	82.61	5.61	4	84.78	8.23	4
		3.94	89.13	2.17	4	93.48	6.52	4	91.30	5.61	4	86.96	6.15	4
		0.98	97.83	5.47	4	100.00	7.53	4	104.35	7.94	4	102.17	5.47	4

P. chrysogenum	393.94	20.12	1.83	4	16.46	4.60	4	25.61	2.11	4	18.29	3.66	4
	98.49	31.10	1.83	4	29.27	2.99	4	32.93	2.11	4	29.27	2.99	4
	19.70	40.24	2.11	4	45.73	3.50	4	43.90	2.99	4	40.24	2.11	4
	3.94	62.20	4.72	4	56.71	4.60	4	56.71	3.50	4	56.71	3.50	4
	0.98	85.98	3.50	4	84.54	9.08	4	73.17	2.99	4	84.15	2.11	4

* n represents the number of replicates.
